# Supplementary material for: Hypertonicity-Affected Genes Are Differentially Expressed in Clear Cell Renal Cell Carcinoma and Correlate with Cancer-Specific Survival
Source: Cancers (Basel). 2019 Dec 18;12(1):6. doi: 10.3390/cancers12010006 (PMC7017076; doi:10.3390/cancers12010006)
Supplement: Supplementary file 1 [file cancers-12-00006-s001.zip › cancers-666681-final-supplementary/cancers-666681-final check-supplementary.docx]

Supplementary Materials

Hypertonicity affected genes are differentially expressed in clear cell renal cell carcinoma and correlate with cancer-specific survival

Siarhei Kandabarau Janna Leiz and Knut Krohn, Stefan Winter, Jens Bedke, Matthias Schwab, Elke Schaeffeler, Bayram Edemir

**Figure S1:** Selection of differentially expressed transcripts affected by hypertonicity in primary cultured inner medullary collecting duct (IMCD) cells either cultivated at 300 or 900 mosmol/kg. Four genes (marked in blue and red) with matched human transcripts/genes were selected for development of the novel OSM-score for prediction of cancer-specific survival in ccRCC patients.


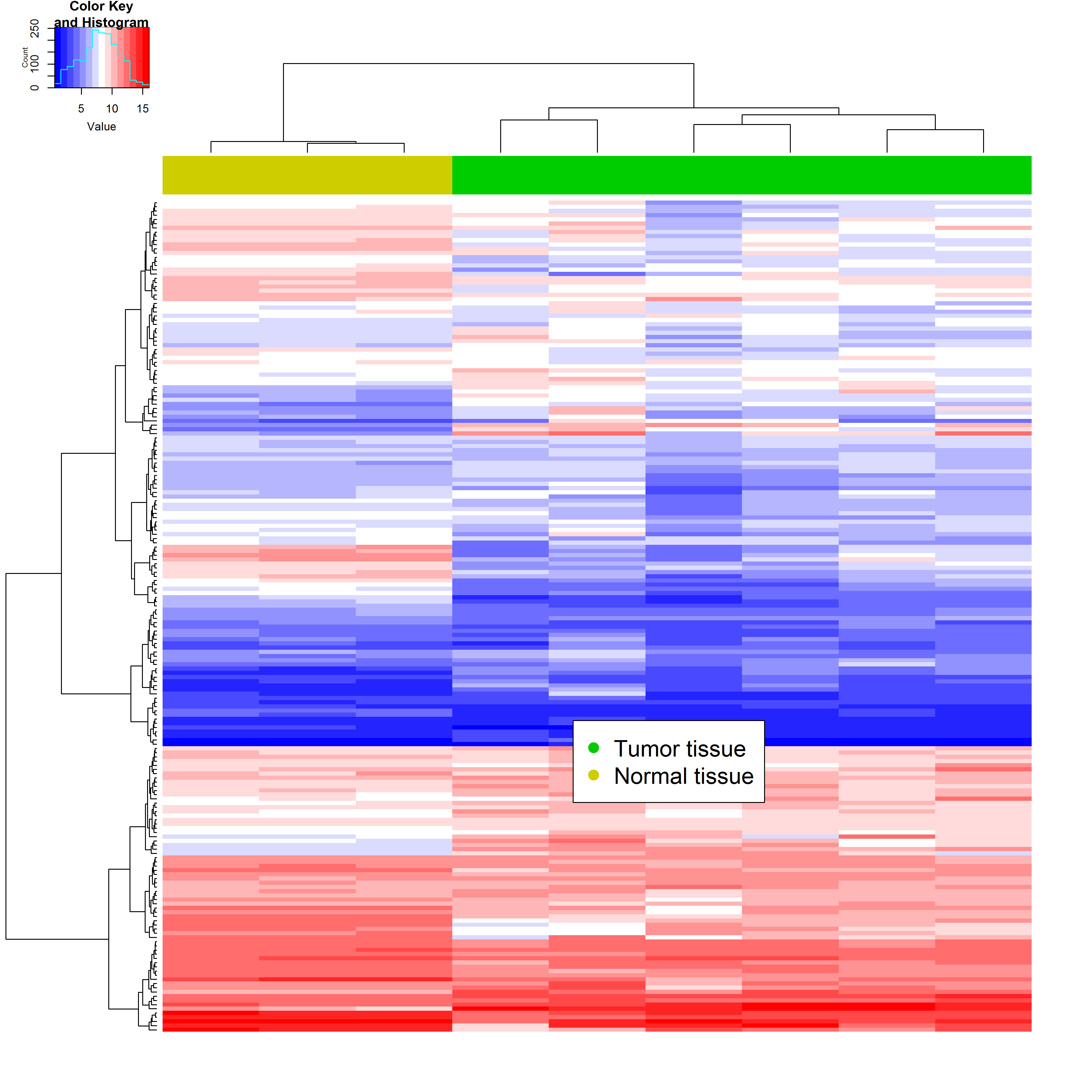


**Figure S2:** Hierarchical clustering of samples from the normal kidney cortex and mouse ccRCCs based on the hypertonicity affected genes. The expression levels were extracted from the supplementary table 1 of Harlander S, et al. (2017). The gene set was able to clearly separate mouse ccRCCs (dark green) from the normal kidney cortex samples (light green).

Uncropped PCR image for Figure 2 A.

**Table S1.** please view at the excel file.

| 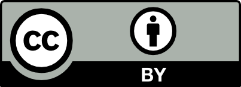 | © 2019 by the authors. Licensee MDPI, Basel, Switzerland. This article is an open access article distributed under the terms and conditions of the Creative Commons Attribution (CC BY) license (http://creativecommons.org/licenses/by/4.0/). |
| --- | --- |
